# Editorial: Reviews in assisted reproduction: 2022

**DOI:** 10.3389/frph.2023.1174047

**Published:** 2023-05-03

**Authors:** Kulvinder Kochar Kaur

**Affiliations:** Reproductive Neuroendocrinology and Infertility, Kulvinder Kaur Centre for Human Reproduction, Jalandhar, India

**Keywords:** assisted reproductive technology, intracytoplasmic sperm injection, heavy metals, vaginal douching agents, telomere length

**Editorial on the Research Topic** Reviews in assisted reproduction: 2022

Since its inception in 1978, IVF has progressively increased in popularity and has helped women conceive in a number of significant ways. An estimated 8–10 million children have been born through IVF and other types of ART. The success rate of these procedures has been determined by several variables, including the mother's age, the cause of her infertility, the fetus' integrity, and her lifestyle.

Recently, we issued an open call for articles on conceptions that followed the use of Assisted reproductive technology (ART), including In vitro fertilization (IVF) and Intracytoplasmic sperm injection (IVF/ICSI).

Out of the five studies that we received, four covered various aspects of enhancing IVF/ICSI outcomes. Of these, three were systematic reviews and meta**-**analyses, while one dealt with the adverse sequelae of IVF over a lengthy period of follow-up in addition to effects on progeny from childhood to adolescence.

The first study, by Huang et al. looked at any advantageous effect of ICSI over IVF in the case of non-male factor infertility. None was observed in terms of pregnancy rates (PR), live birth rates (LBR), or ongoing pregnancy rates (OPR).

Heavy metals and metalloids are recognized as having greater atomic numbers and densities that are five times greater than the density of water and are usually categorized into essential metals (manganese, copper, chromium, zinc, selenium, etc.) and non-essential metals (lead, cadmium, mercury, etc). Essential metals and metalloids have significant benefits in the management of human health and that of other living organisms at bio-permissible levels established by standard regulatory bodies. These include manganese, copper, chromium, zinc, selenium, and others, some of which are key components of numerous metabolic controlling enzymes and signaling pathways. Nevertheless, once levels exceed the regulatory limit, biotoxicity affecting different organs and systems occurs in biological systems. The metals and metalloids classified as non-essential heavy metals and metalloids are toxic even at trace levels. These include lead, arsenic, cadmium, chromium, mercury, etc. Obasi et al.'s 16 articles documented the correlations between specific heavy metals and IVF outcomes, while 14 articles summarized the role of heavy metals in reproductive issues. For the studies on IVF outcomes, various human samples were analyzed for heavy metals. Heavy metals and metalloids (Pb, Hg, Cd, Cr, Mn, As) were negatively associated with oocyte fertilization/pregnancy rates in hair, follicular fluid, serum, urine, and seminal plasma samples, while Cd and Hg showed no correlations in whole blood samples. For the studies on reproductive concerns, high concentrations of heavy metals/metalloids were implicated in the following conditions: infertility (Cd, Pb, Ba, U), spontaneous abortion/miscarriage (B, c, Sb), congenital heart disease (Al, Mg, Cd), PCOS (As, Cd, Hg, Pb), endometriosis (Pb), and uterine leiomyomata (Hg).

The incidence of oocyte retrieval-associated pelvic infection (OPU-PI) has been documented to be 0.02% according to the ESHRE results, while other studies documented an incidence rate of up to 0.3%–1.5%. It is widely acknowledged that pre-operative vaginal preparation still cannot prevent infection in all cases because every possible action is taken to minimize the risk of pelvic infection. Nevertheless, the use of antiseptic solutions for vaginal douching prior to OPU in ART is controversial. Two major issues with the use of antiseptic solutions in patients who are actively undergoing OPU with *in vitro* fertilization (IVF) are the benefits in reducing complications associated with OPU in addition to the toxicity impact on oocyte quality.

The aim of the study by Meng et al. was to assess the effects of six vaginal douching agents (iodine, saline, iodine followed by saline, chlorhexidine acetate followed by saline, ozone, and potassium permanganate) on oocyte retrieval-associated pelvic infection (OPU-PI) and IVF outcome in patients who underwent ART. Eight studies were included, with a total sample size of 12,567. The results of this network meta-analysis showed that ozone can significantly decrease OPU-PI, iodine followed by saline can be the most effective antiseptic protocol without affecting the quality of oocytes, and chlorhexidine acetate followed by saline can increase the clinical pregnancy rate of patients. In summary, this review documents the best available evidence regarding the use of douching prior to ART.

The fourth study, by Hart and Wijs, looked at long-term effects on children. Hart has been one of the initiators of the Western Australian Growing Up Healthy Study (WAGUHS), which looked at children born after IVF/ICSI. Maximum results are typically secondary to the correlated elevated risk of pregnancy-induced hypertension (PIH) and preeclampsia in women who have conceived through ART. A significant, correlated problem associated with IVF/ICSI is the epigenetic alterations in children, in particular, DNA methylation; however, studies from WAGUHS have shown that the majority of these epigenetic changes are reversed by adulthood. However, in the past 23 years, greater emphasis has been placed on ICSI, and less of this study has included ICSI cohorts for better comparison of IVF/ICSI rather than Reines cohort (population-based for Western Australia). Furthermore, findings from the long-term follow-up and quality of life study conducted by researchers in Melbourne based on childhood hospitalization data from the United Kingdom and the data published from the WAGUHS will aid in reassuring prospective parents who might need ART to conceive.

**Figure 1:**
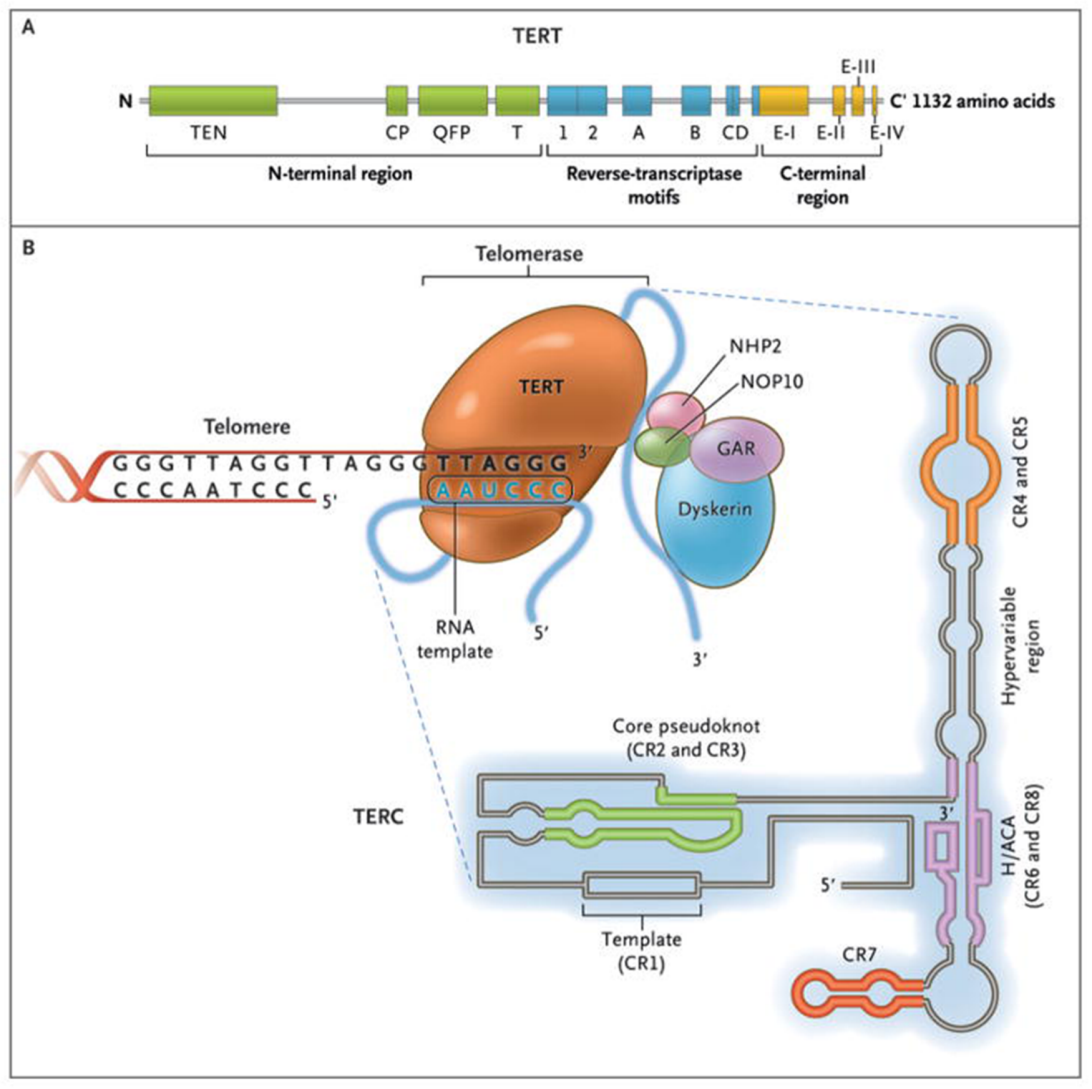
The Telomerase Complex and Its Components. The enzyme telomerase reverse transcriptase (TERT), its RNA component (TERC), the protein dyskerin, and other associated proteins (NHP2, NOP10, and GAR1) are shown. Telomerase catalytically adds TTAGGG hexameric nucleotide repeats to the 3′-hydroxyl end of the telomeric leading strand, using a specific sequence in the RNA component as the template. TERT contains three major domains: the N-terminal region, the reverse-transcriptase motifs, and the C-terminal region, all containing evolutionarily conserved motifs. TERC contains 451 nucleotides in seven conserved regions (CR1 through CR7), including the template (CR1), a (CR1 through CR7), including the template (CR1), and an H/ACA box, a hairpin nucleotide sequence characteristic of a class of small nucleolar RNAs involved in RNA processing. Image Courtesy of Calado RT, Young NS (1).

Telomere length was the subject of the fifth study, by Moustakiel et al. The correlation between sperm telomere length and mitochondrial capacity, including its structure and functions, is poorly understood. Mitochondria are structurally and functionally separate organelles located in the midpiece of the spermatozoon. Mitochondria generate adenosine triphosphate (ATP) through oxidative phosphorylation (OXPHOS), which is necessary for sperm motility, and produce reactive oxygen species (ROS). While a moderate quantity of ROS is key for egg—sperm fusion and fertilization, increased ROS production is primarily associated with telomere shortening, sperm DNA fragmentation, and changes in methylation patterns leading to male infertility. The aim of their review was to emphasize the functional connection between mitochondrial biogenesis and telomere length in male infertility, as mitochondrial lesions have a damaging impact on telomere length, leading to both telomere lengthening and reprogramming of mitochondrial biosynthesis. Moreover, the authors tried to show how both inositol and antioxidants can positively affect male fertility, despite some recent reports that antioxidants have no role in male infertility (see Figure 1 on the telomere complex and its components).
